# Targeted C‐to‐T Base Editing in the *Arabidopsis* Plastid Genome

**DOI:** 10.1002/cpz1.70075

**Published:** 2025-01-06

**Authors:** Issei Nakazato, Shin‐ichi Arimura

**Affiliations:** ^1^ Laboratory of Plant Molecular Genetics, Graduate School of Agricultural and Life Sciences The University of Tokyo Tokyo Japan; ^2^ Japan Society for the Promotion of Science Tokyo Japan

**Keywords:** *Arabidopsis thaliana*, genome editing, plastid genome

## Abstract

*Arabidopsis thaliana*, particularly the ecotype Columbia‐0 (Col‐0), has been extensively employed in the study of genetics of the nuclear genome. However, the difficulty of modifying the plastid genome of Col‐0, the most widely used ecotype, has hindered investigation of the functional interactions between nuclear‐encoded and plastid‐encoded genes in this ecotype. Recently, we achieved targeted base editing, substituting a specific C:G pair with a T:A pair in the plastid genome of Col‐0 through the application of genome‐editing technology. This article introduces the method employed to accomplish this targeted base editing. The process involves four steps: (i) designing and constructing a binary vector encoding the genome‐editing enzyme, (ii) introducing the binary vector into the nuclear genome of Col‐0 via floral dipping, (iii) identifying base‐edited plants, and (iv) verifying inheritance of the edited base(s) by the next generation. © 2025 The Author(s). Current Protocols published by Wiley Periodicals LLC.

**Basic Protocol 1**: Design and construction of a binary vector encoding ptpTALECD or ptpTALECD_v2

**Basic Protocol 2**: *Agrobacterium*‐mediated introduction of a binary vector into the *Arabidopsis* nuclear genome

**Basic Protocol 3**: Selection of plants harboring T‐DNA in the nucleus and detection of base editing in the plastid genome

## INTRODUCTION

**Figure 1:**
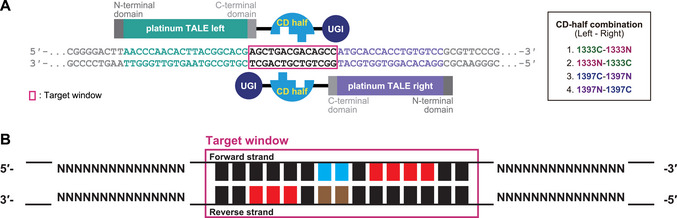
C:G‐to‐T:A base editing by ptpTALECD. (**A**) Schematic image of ptpTALECD and its target sequence. CD is split after Gly 1333 or Gly 1397, and its N‐ and C‐terminal halves are fused to the left or right TALE domain, respectively. In the right panel, “1333C‐1333N” indicates that the CD domain is split after Gly 1333, with the C‐ and N‐terminal halves fused to the left and right TALEs, respectively. The same convention applies to the remaining constructs. CD has deamination activity when the split halves meet. Uracil glycosylase inhibitor (UGI), fused after CD halves, facilitates C:G‐to‐T:A base editing by inhibiting removal of uracil that is generated from cytosine by CD. Platinum TALE is the TALE of platinum TALEN. Modified from Nakazato et al. (2021). (**B**) Distinct patterns of the position of edited cytosines in the target window. When CD is split after Gly 1333, cytosines near the center of the target window are preferentially edited. Specifically, forward‐strand cytosines (indicated in cyan) and reverse‐strand cytosines (indicated in brown) tend to be edited by 1333N‐1333C and 1333C‐1333N, respectively. Alternatively, when CD is split after Gly 1397, cytosines near the right side of the forward strand and the left side of the reverse strand in the target window (indicated in red) are likely to be edited. “NN…NN” indicates the TALE‐binding sequences.

*Arabidopsis thaliana* has served as a model organism for plant research, particularly in the field of biology of dicots, for several decades. Four reasons underscore the suitability of this species as a model plant. First, it has a short life cycle, namely, approximately 2 to 3 months. Second, its small size allows for the cultivation of a large number of individuals per unit area or volume. Third, its genome was sequenced by the year 2000, and its nuclear genome is relatively small (∼120 Mb) (Cheng et al., 2017) and diploid. Fourth, foreign genes can be introduced into the nuclear genome through a relatively simple method known as floral dipping (Clough & Bent, 1998). These characteristics are particularly beneficial for genetics research, especially concerning nuclear‐encoded genes. Conversely, *A. thaliana* has not been extensively utilized in plastid genetics, primarily due to the technical challenges of modifying its plastid genome. The plastid genome of *A. thaliana*, as in many other plant species, is maternally inherited (Martinez et al., 1997), precluding recombination via mating. Consequently, artificial modification of the plastid genome serves as a powerful tool for analyzing and enhancing traits regulated by plastid‐encoded genes. The first successful targeted modification of the plastid genome in terrestrial plants, through the introduction of foreign genes into specific loci—commonly referred to as plastid transformation—was reported in 1990 (Svab et al., 1990) and has since been applied in more than 20 species, with tobacco as the primary model (Liu et al., 2023). In *A. thaliana*, the generation of the first transplastomic plants, with foreign genes integrated into the plastid genome, was achieved in 1998, although these plants were sterile (Sikdar et al., 1998). In the late 2010s, fertile transplastomic plants were obtained using a knockout (KO) mutant of the *ACC2* gene, though the transformation efficiency in wild‐type *A. thaliana* remained very low and has not yet been improved (Ruf et al., 2019; Yu et al., 2017). The wild‐type *A. thaliana* ecotype Columbia‐0 (Col‐0) currently represents one of the most well‐characterized plants in nuclear genome genetics. Successful plastid genome modification in Col‐0 would significantly advance research not only on plastid‐encoded gene functions but also on the functional interactions between nuclear and plastid genomes. By applying the genome‐editing enzyme known as DddA‐derived cytosine base editor, which was originally designed to edit human mitochondrial DNA (Mok et al., 2020), C:G pairs in the plastid genome of Col‐0 were substituted with T:A pairs (Nakazato et al., 2021). Instead of employing the CRISPR/Cas system, we utilized a protein‐only genome‐editing enzyme based on transcription activator–like effector nuclease (TALEN), named plastid‐targeted platinum TALECD (ptpTALECD). One important reason to use a protein‐only genome‐editing enzyme is the inefficiency of transporting a guide RNA, an essential component of the CRISPR/Cas system, into plastids. In contrast, proteins can be transported into plastids by fusing a plastid‐targeting signal sequence to their N‐terminus (Li & Chiu, 2010). ptpTALECD is a bimolecular enzyme composed of two distinct molecules. The first molecule contains the plastid‐targeting signal of *At*RECA1 [PTP (Cerutti et al., 1992; Cao et al., 1997)]; the DNA‐binding domain of platinum TALEN [TALE (Sakuma et al., 2013)]; the C‐terminal half of split DddA, a cytidine deaminase (CD) that operates on double‐stranded DNA (dsDNA) (Mok et al., 2020); and uracil glycosylase inhibitor [UGI (Komor et al., 2016)]. The other molecule consists of PTP, TALE, the N‐terminal half of split DddA, and UGI (Fig. 1A). TALEs specifically bind to their target sequence, ranging from 6 to 21 bp, and specific C:G pairs within this target window, the sequence in between the TALE binding sequences, are substituted with T:A pairs. Initially, the sequences that the TALEs would bind to were designed, and subsequently, a binary vector encoding ptpTALECD was constructed. This vector was introduced into the nuclear genome of Col‐0 via floral dipping. We succeeded in generating plants in which only C:G(s) in the target window were edited. In addition, this editing occurred across all copies of the plastid genome, which exists in hundreds to thousands of copies per cell (Zoschke et al., 2007); in other words, homoplasmy was achieved. Furthermore, we obtained homoplasmically base‐edited plants that were unlikely to contain foreign genes (Nakazato et al., 2021). In these plants, base edits are fixed in homoplasmy, and no additional mutations will be introduced, allowing for a clearer analysis of the relationship between the introduced mutation(s) and phenotype. Subsequently, a modified version of ptpTALECD (ptpTALECD_v2) containing a high‐activity CD (DddA11′) was developed, and it was easier to replace C on the 3′ side of A, T, C, and G compared to in ptpTALECD (Nakazato et al., 2023). ptpTALECD and ptpTALECD_v2 are expected to facilitate genetic study of the plastid genome in Col‐0, addressing a longstanding challenge.

This article outlines the methodology for targeted C:G‐to‐T:A base editing in the plastid genome of Col‐0 using ptpTALECD and ptpTALECD_v2. The procedure involves four key steps: (i) designing and constructing binary vectors encoding ptpTALECD or ptpTALECD_v2, (ii) introducing the binary vectors into the nuclear genome of Col‐0 via floral dipping (nuclear transformation), (iii) verifying base substitutions in the target window of T_1_ plants, and (iv) confirming the inheritance of the substituted base in the next generation (T_2_ plants). In Basic Protocol 1, the procedure of vector design and construction is outlined. In Basic Protocol 2, we describe the methods for cultivating Col‐0 and introducing the vector into its nuclear genome. Lastly, in Basic Protocol 3, we detail the approach for determining the genotypes of the transformants.

## Basic Protocol 1 DESIGN AND CONSTRUCTION OF A BINARY VECTOR ENCODING ptpTALECD or ptpTALECD_v2

In this protocol, we describe how to design TALE binding sequences for your target and how to construct a binary vector encoding the base editors. The efficiency of base editing is influenced by the design of the TALE binding sequences and the selection of ptpTALECD or ptpTALECD_v2. Here, key considerations to optimize these processes are outlined. In DdCBE and ptpTALECD, DddA is split after Gly 1333 or Gly 1397 to reduce its cell toxicity, and its N‐ and C‐terminal halves are separately fused to TALEs (Fig. 1A). In ptpTALECD_v2, DddA11′ is split at the same positions, and its N‐ and C‐terminal halves are fused to TALEs. Mok et al. demonstrated that the positions of cytosines edited in the target window (14 to 18 bp) of human mitochondrial genomes by DdCBE depend on the split site of DddA (Fig. 1B). When DddA was split after Gly 1333, cytosines in the center of the target window (highlighted in cyan and brown in Fig. 1B) were predominantly edited. On the other hand, when DddA was split after Gly 1397, cytosines in the left side of the forward strand of dsDNA and the right side of the reverse strand of dsDNA (highlighted in red in Fig. 1B) were predominantly edited (Mok et al., 2020). The positions of cytosines edited by ptpTALECD were similar to those in human mitochondria (Nakazato et al., 2021, 2023). Both ptpTALECD and ptpTALECD_v2 could homoplasmically edit TCs and ACs. On the other hand, ptpTALECD_v2 could homoplasmically edit GCs and CCs, some of which were not homoplasmically edited by ptpTALECD. However, ptpTALECD_v2 edited more cytosines in the target window and more frequently introduced off‐target mutations in the plastid genome compared to ptpTALECD (Nakazato et al., 2023). Based on these results, it is preferable to use ptpTALECD for targeting TC or AC, whereas ptpTALECD_v2 is more suitable for targeting GC or CC.

Designing TALE binding sequences that ensure specific binding to the appropriate sequences is crucial for minimizing off‐target effects. The plastid genome of *A. thaliana* is significantly smaller than the nuclear genome, consisting of only 154 kb (Sato et al., 1999). Consequently, it is generally feasible to achieve precise target recognition without off‐target effects when each TALE binding sequence is ∼10 bp in length; however, to further minimize off‐target effects, it is recommended to design each TALE‐binding sequence to be as long as possible (up to 21 bp).

**Figure 2:**
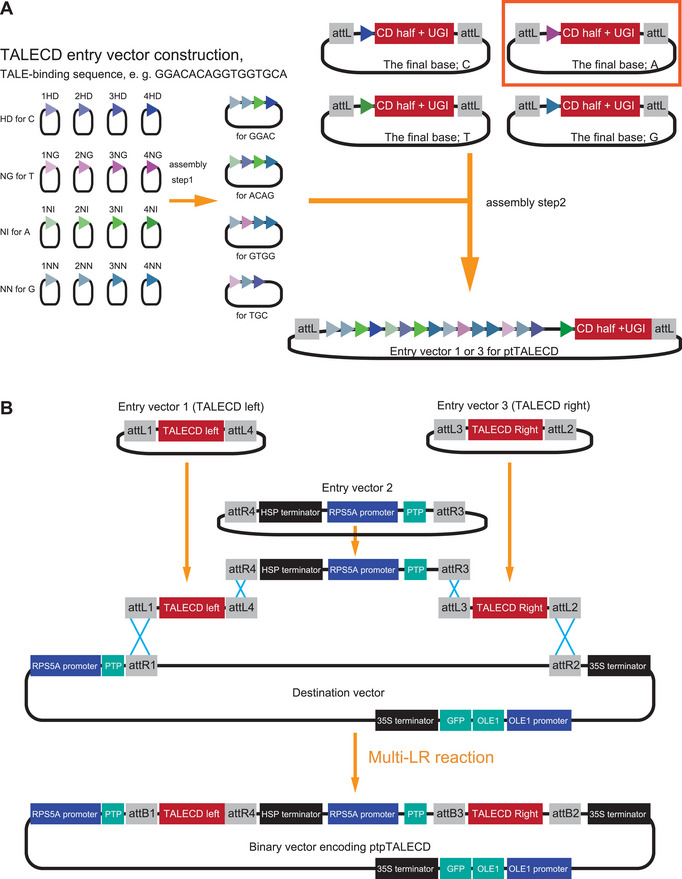
The strategy for constructing ptpTALECD (or ptpTALECD_v2)‐encoding binary vectors. (**A**) Assembly Steps 1 and 2 for constructing TALECD open reading frames. When the  12^th^ and 13^th^ amino acids of each TAL repeat are HD, NG, NI, and NN, the TAL repeat preferentially binds to C, T, A, and G, respectively. In Assembly Step 1, 1 to 4 modules each encoding a TAL repeat are assembled into a single plasmid. In Assembly Step 2, the plasmids are further assembled into a single plasmid encoding the left or right hand of TALECD. In this step, the choice of vector (shown in the upper right) depends on the final base of the TALE‐binding sequence. (**B**) Schematic image of Assembly Step 3. The binary vector encoding ptpTALECD or ptpTALECD_v2 is generated via multi‐LR reaction from four plasmids. Modified from Nakazato et al. (2021).

The binary vector is generated through a three‐step construction process (Fig. 2). This vector, a single large Ti‐plasmid (∼22 kb), encodes a pair of ptpTALECD (or ptpTALECD_v2) molecules. Consequently, unlike many experiments using TALEN or TALEN‐based enzymes, this approach eliminates the need to co‐transform two vectors, each encoding one of the two TALEN (or TALE‐based enzyme) molecules. The first step of the construction process (hereafter called Assembly Step 1) involves assembling TALE components by fusing up to four TAL repeats, each recognizing a single nucleotide (denoted by the triangle symbol in Fig. 2A, left). This assembly is performed according to the protocol provided with the Platinum Gate TALEN Kit from Addgene [see Platinum Gate assembly ‐Step 1‐ (Sakuma et al., 2013)]. The second step (hereafter called Assembly Step 2) involves using 3 to 5 plasmids generated in Assembly Step 1 to assemble a TALE domain that can recognize a desired 6‐ to 21‐nucleotide sequence. This domain is simultaneously inserted into the vector (depicted in the upper right of Fig. 2A), which encodes a TAL repeat recognizing the final nucleotide in the TALE‐binding sequence, along with a CD half, UGI, and N‐ or C‐termini of the TALE domain. This insertion is accomplished in a single reaction using the Golden Gate assembly method, resulting in a vector that encodes one of the ptpTALECD molecules and is compatible with the subsequent multi‐LR reaction. For this step, instead of using the ptCMV‐153/47‐VR‐(HD, NG, NI, NN) or ptCMV‐136/63‐VR‐(HD, NG, NI, NN) vectors provided in Platinum Gate TALEN Kit, plasmids (Addgene, cat. no. 171724‐171735, 191579‐191598, 191603‐191614, and 199797‐199804) are used. To construct vectors encoding ptpTALECD_v2, utilize the vectors labeled with “DddA11'” (Addgene, cat. no. 191603‐191606, 191611‐191614, and 199797‐199804). In the third step (hereafter called Assembly Step 3), through a multi‐LR reaction with LR Clonase™ II Plus (Thermo Fisher Scientific), the destination vector, pDest_pK7WG2_pRPS5A_CTP OleGFP for ptpTALECD (Addgene, cat. no. 171723), is combined with three entry vectors: the two vectors generated in Assembly Step 2 and an intermediate vector, E2_pENTR_R4R3_T CTP for ptpTALECD (Addgene, cat. no. 171736) (Fig. 2B). This assembly results in a binary vector that encodes both open reading frames (ORFs) of ptpTALECD with plastid transit peptides.

### Materials

Platinum Gate TALEN Kit (Addgene, cat. no. 1000000043)
rCutSmart^TM^ Buffer (New England Biolabs, cat. no. B6004S)
BsaI‐HFv2 (New England Biolabs, cat. no. R3733L)
Competent Quick DH5a (TOYOBO, cat. no. DNA‐913F)
SOC medium (e.g., that included with *E. coli* HST08 Premium Competent Cells, TaKaRa, cat. no. 9128)
0.1 M IPTG solution (see recipe)
20 mg ml^−1^ X‐Gal solution (see recipe)
LB spectinomycin plate (see recipe)
LB spectinomycin medium (see recipe)
Plasmid extraction kit (e.g., Nippon Genetics, cat. no. FG‐90502)
Primers:
  pCR8‐F1, 5′‐ TTGATGCCTGGCAGTTCCCT ‐3′
  TAL‐F1, 5′‐ TTGGCGTCGGCAAACAGTGG ‐3′
  TAL‐R2, 5′‐ GGCGACGAGGTGGTCGTTGG ‐3′
  TAL‐F2, 5′‐ GGAGGCAGTGCATGCATGGC ‐3′
  pK7WG2_fsFw, 5′‐ TTCTCTTAGGTTTACCCGCC ‐3′
  tHSP_fsRv, 5′‐ CCATAGTCCATACCATAGCAC ‐3′
  Ole_fsRv, 5′‐ CTAAGTAGGGTGCCGGGGAT ‐3′
  1333N_checkRv, 5′‐ GGCAGGCAATTGAGGAGCTG ‐3′
  1333C_checkRv, 5′‐ GGCGTAATTCGGATACGGAG ‐3′
  1397N_checkRv, 5′‐ CAGGCAATTGTGGAGCTGAG ‐3′
  1397N_TtoC_Rv, 5′‐ GGGCTGATTGACCTTCAACAT ‐3′
  1397C_checkRv, 5′‐ CGTTTCTCCTGTAGCTCCTC ‐3′
E1 and E3 vectors (Table 1)
TE, pH 8.0 (Nippon Gene, cat. no. 314‐90021)
Tango Buffer (10×) (Thermo Fisher Scientific, cat. no. BY5)
10 mM dithiothreitol (see recipe)
BsmBI (Thermo Fisher Scientific, cat. no. ER0452)
LB kanamycin plate (see recipe)
Sterile distilled water (e.g., Nippon Gene, cat. no. 312‐90103)
KOD One^®^ PCR Master Mix ‐Blue‐ (dye‐containing 2× PCR master mix; TOYOBO, cat. no. KMM‐201)
250 bp DNA Ladder (Dye Plus) (TaKaRa, cat. no. 3424A)
1% (w/v) agarose gel (see recipe)
1× TBE buffer (see recipe)
LB kanamycin medium (see recipe)
LR Clonase™ II Plus (with proteinase K included; Thermo Fisher Scientific, cat. no. 12538200)
*E. coli* HST08 Premium Competent Cells (TaKaRa, cat. no. 9128)
1 kb DNA Ladder (Dye Plus) (TaKaRa, cat. no. 3426A)
Gel Loading Dye, Purple (6×) (New England Biolabs, cat. no. B7024S)
Sequencing primers for binary vectors (see Table 2)

8‐strip 0.2‐ml PCR tubes (e.g., Nippon Genetics, cat. no. FG‐028DC)
Thermal cycler (e.g., Applied Biosystems^TM^ MiniAmp Plus, Thermo Fisher Scientific)
1.5‐ml tubes (e.g., INA OPTICA, cat. no. CF‐0150)
1.5‐ml tube incubator (e.g., Cool Thermo Unit, CTU‐Neo, TAITEC), 37°C and 42°C
Bacteria spreader (e.g., AS ONE, cat. no. 1‐3192‐01)
Bacterial incubator, 37°C
14‐ml culture tubes (e.g., Corning Life Sciences, cat. no. 352059)
Autoclaved bamboo skewer or similar implement
Shaking incubator (e.g., Bioshaker, TAITEC, cat. no. BR‐43FL)
Centrifuge (e.g., TOMY, cat. no. MDX310F)
Spectrophotometer (NanoDrop One^C^, Thermo Fisher Scientific)
Sequence analysis software (e.g., Geneious Prime)
Mupid‐2plus system (TaKaRa)
Gel Doc EZ System (Bio‐Rad) with compatible PC

Additional reagents and equipment for preparing master mixes (see Tables 3 and 4) and restriction enzyme digest (see Table 6) and for Sanger sequencing and agarose gel electrophoresis (see Current Protocols article: Voytas, 2000)

**Table 1:** Plasmids Employed to Assemble a Binary Vector Encoding ptpTALECD or ptpTALECD_v2

Name	Addgene ID	Left/Right	Final base of TALE binding sequence	Base editor	Assembly Step
E1_pENTR_L1‐L4_HD_G1333‐DddtoxA‐C	191579	Left	C	ptpTALECD	2
E1_pENTR_L1‐L4_HD_G1333‐DddtoxA‐N	191581	Left	C	ptpTALECD	2
E1_pENTR_L1‐L4_HD_G1397‐DddtoxA‐C	191583	Left	C	ptpTALECD	2
E1_pENTR_L1‐L4_HD_G1397‐DddtoxA‐N	191585	Left	C	ptpTALECD	2
E1_pENTR_L1‐L4_NG_G1333‐DddtoxA‐C	191580	Left	T	ptpTALECD	2
E1_pENTR_L1‐L4_NG_G1333‐DddtoxA‐N	191582	Left	T	ptpTALECD	2
E1_pENTR_L1‐L4_NG_G1397‐DddtoxA‐C	191584	Left	T	ptpTALECD	2
E1_pENTR_L1‐L4_NG_G1397‐DddtoxA‐N	191586	Left	T	ptpTALECD	2
E1_pENTR_L1‐L4_NI_G1333‐DddtoxA‐C	171724	Left	A	ptpTALECD	2
E1_pENTR_L1‐L4_NI_G1333‐DddtoxA‐N	171725	Left	A	ptpTALECD	2
E1_pENTR_L1‐L4_NI_G1397‐DddtoxA‐C	171726	Left	A	ptpTALECD	2
E1_pENTR_L1‐L4_NI_G1397‐DddtoxA‐N	171727	Left	A	ptpTALECD	2
E1_pENTR_L1‐L4_NN_G1333‐DddtoxA‐C	171728	Left	G	ptpTALECD	2
E1_pENTR_L1‐L4_NN_G1333‐DddtoxA‐N	171729	Left	G	ptpTALECD	2
E1_pENTR_L1‐L4_NN_G1397‐DddtoxA‐C	171730	Left	G	ptpTALECD	2
E1_pENTR_L1‐L4_NN_G1397‐DddtoxA‐N	171731	Left	G	ptpTALECD	2
E3_pENTR_L3‐L2_HD_G1333‐DddtoxA‐C	191587	Right	C	ptpTALECD	2
E3_pENTR_L3‐L2_HD_G1333‐DddtoxA‐N	191590	Right	C	ptpTALECD	2
E3_pENTR_L3‐L2_HD_G1397‐DddtoxA‐C	191593	Right	C	ptpTALECD	2
E3_pENTR_L3‐L2_HD_G1397‐DddtoxA‐N	191596	Right	C	ptpTALECD	2
E3_pENTR_L3‐L2_NG_G1333‐DddtoxA‐C	171732	Right	T	ptpTALECD	2
E3_pENTR_L3‐L2_NG_G1333‐DddtoxA‐N	171733	Right	T	ptpTALECD	2
E3_pENTR_L3‐L2_NG_G1397‐DddtoxA‐C	171734	Right	T	ptpTALECD	2
E3_pENTR_L3‐L2_NG_G1397‐DddtoxA‐N	171735	Right	T	ptpTALECD	2
E3_pENTR_L3‐L2_NI_G1333‐DddtoxA‐C	191588	Right	A	ptpTALECD	2
E3_pENTR_L3‐L2_NI_G1333‐DddtoxA‐N	191591	Right	A	ptpTALECD	2
E3_pENTR_L3‐L2_NI_G1397‐DddtoxA‐C	191594	Right	A	ptpTALECD	2
E3_pENTR_L3‐L2_NI_G1397‐DddtoxA‐N	191597	Right	A	ptpTALECD	2
E3_pENTR_L3‐L2_NN_G1333‐DddtoxA‐C	191589	Right	G	ptpTALECD	2
E3_pENTR_L3‐L2_NN_G1333‐DddtoxA‐N	191592	Right	G	ptpTALECD	2
E3_pENTR_L3‐L2_NN_G1397‐DddtoxA‐C	191595	Right	G	ptpTALECD	2
E3_pENTR_L3‐L2_NN_G1397‐DddtoxA‐N	191598	Right	G	ptpTALECD	2
E1_pENTR_L1‐L4_HD_G1397‐DddA11'‐C	191603	Left	C	ptpTALECD_v2	2
E1_pENTR_L1‐L4_HD_G1333‐DddA11'‐C	199797	Left	C	ptpTALECD_v2	2
E1_pENTR_L1‐L4_NG_G1397‐DddA11'‐C	191604	Left	T	ptpTALECD_v2	2
E1_pENTR_L1‐L4_NG_G1333‐DddA11'‐C	199798	Left	T	ptpTALECD_v2	2
E1_pENTR_L1‐L4_NI_G1397‐DddA11'‐C	191605	Left	A	ptpTALECD_v2	2
E1_pENTR_L1‐L4_NI_G1333‐DddA11'‐C	199799	Left	A	ptpTALECD_v2	2
E1_pENTR_L1‐L4_NN_G1397‐DddA11'‐C	191606	Left	G	ptpTALECD_v2	2
E1_pENTR_L1‐L4_NN_G1333‐DddA11'‐C	199800	Left	G	ptpTALECD_v2	2
E3_pENTR_L3‐L2_HD_G1397‐DddA11'‐N	191611	Right	C	ptpTALECD_v2	2
E3_pENTR_L3‐L2_HD_G1333‐DddA11'‐N	199801	Right	C	ptpTALECD_v2	2
E3_pENTR_L3‐L2_NG_G1397‐DddA11'‐N	191612	Right	T	ptpTALECD_v2	2
E3_pENTR_L3‐L2_NG_G1333‐DddA11'‐N	199802	Right	T	ptpTALECD_v2	2
E3_pENTR_L3‐L2_NI_G1397‐DddA11'‐N	191613	Right	A	ptpTALECD_v2	2
E3_pENTR_L3‐L2_NI_G1333‐DddA11'‐N	199803	Right	A	ptpTALECD_v2	2
E3_pENTR_L3‐L2_NN_G1397‐DddA11'‐N	191614	Right	G	ptpTALECD_v2	2
E3_pENTR_L3‐L2_NN_G1333‐DddA11'‐N	199804	Right	G	ptpTALECD_v2	2
pDest_pK7WG2_pRPS5A_CTP OleGFP	171723	N/A	N/A	Both	3
E2_pENTR_R4R3_T CTP	171736	N/A	N/A	Both	3

**Table 2:** Sequencing Primers for Binary Vectors

		Primer name			
Site of CD split	Base editor	1	2	3	4
Gly1333	ptpTALECD	Ole_fsRv	tHSP_fsRv	1333N_checkRv	1333C_checkRv
Gly1333	ptpTALECD_v2	Ole_fsRv	tHSP_fsRv	1333N_checkRv	1397N_TtoC_Rv
Gly1397	ptpTALECD	Ole_fsRv	tHSP_fsRv	1397N_checkRv	1397C_checkRv
Gly1397	ptpTALECD_v2	Ole_fsRv	tHSP_fsRv	1397N_checkRv	1397C_checkRv

**Table 3:** Composition of the Reaction Mixture for the Golden Gate Reaction in Assembly Step 1*^a^*

	Number of modules			
	1	2	3	4
pFUS2 vector (25 ng µl^−1^; from Platinum Gate TALEN Kit, Addgene, cat. no. 1000000043)	0.3 µl	0.3 µl	0.3 µl	0.3 µl
Module (50 ng µl^−1^; from Platinum Gate TALEN Kit, Addgene, cat. no. 1000000043)	0.3 µl	0.3 µl each	0.3 µl each	0.3 µl each
10× T4 DNA Ligase Reaction Buffer (New England Biolabs, cat. no. B0202S)	0.2 µl	0.2 µl	0.2 µl	0.2 µl
Quick Ligase (from Quick Ligation^TM^ Kit, New England Biolabs, cat. no. M2200L)	0.1 µl	0.1 µl	0.1 µl	0.1 µl
BsaI‐HFv2 (New England Biolabs, cat. no. R3733L)	0.1 µl	0.1 µl	0.1 µl	0.1 µl
Sterile distilled water (e.g., Nippon Gene, cat. no. 312‐90103)	0.9 µl	0.6 µl	0.3 µl	0 µl
Total	2.0 µl	2.0 µl	2.0 µl	2.0 µl

^*a*^ Modified from the Platinum Gate TALEN construction protocol (https://www.addgene.org/kits/yamamoto‐platinumgate/).

**Table 4:** Composition of the Reaction Mixture for the Golden Gate Reaction in Assembly Step 2*^a^*

	Number of modules			
	1	2	3	4
E1 or E3 vector (50 ng µl^−1^; see Table 1)	0.3 µl	0.3 µl	0.3 µl	0.3 µl
pFUS2 vector (50 ng µl^−1^; from Platinum Gate TALEN Kit, Addgene, cat. no. 1000000043)	0.6 µl	0.6 µl each	0.6 µl each	0.6 µl each
10× T4 DNA Ligase Reaction Buffer (New England Biolabs, cat. no. B0202S)	0.4 µl	0.4 µl	0.4 µl	0.4 µl
Quick Ligase (from Quick Ligation^TM^ Kit, New England Biolabs, cat. no. M2200L)	0.2 µl	0.2 µl	0.2 µl	0.2 µl
BsmBI (Thermo Fisher Scientific, cat. no. ER0452)	0.2 µl	0.2 µl	0.2 µl	0.2 µl
Sterile distilled water (e.g., Nippon Gene, cat. no. 312‐90103)	1.7 µl	1.1 µl	0.5 µl	0
Total	4.0 µl	4.0 µl	4.0 µl	4.0 µl

^*a*^ Modified from the Platinum Gate TALEN construction protocol (https://www.addgene.org/kits/yamamoto‐platinumgate/).

### Design of TALE binding sequences

1 Based on the positional tendency of edited cytosines in the target window (Fig. 1B), design the location of the target window, the split site of DddA, and the position of the target base within the target window to facilitate base substitution.
2 Design sequences (6 to 21 bp each) to which two TALE domains can bind. Additionally, ensure that, as much as possible, the TALE begins binding at the base located one nucleotide downstream of a thymine.

### Vector construction

#### Assembly Step 1

**Figure 3:**
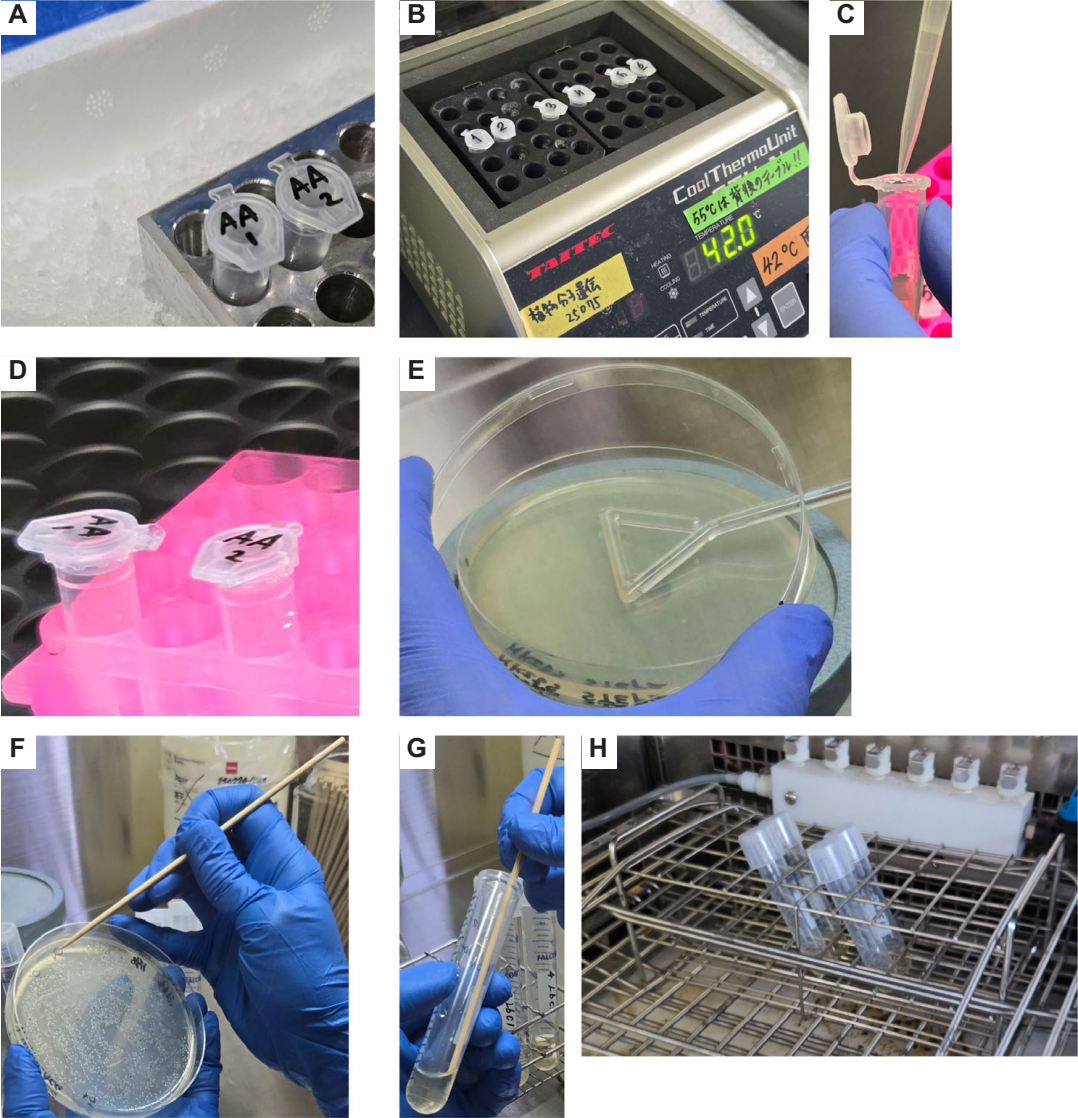
Procedures for *Escherichia coli* transformation and culture. (**A**) Incubation on ice for 30 min following the addition of reaction mixture to dispensed *E. coli*. (**B**) Heat shock at 42°C for 45 s following the incubation on ice. (**C**) Addition of SOC medium to heat‐shocked *E. coli*. (**D**) Incubation at 37°C for 1 hr following the addition of SOC medium. (**E**) Plating of incubated *E. coli* onto an LB solid plate containing antibiotics. (**F**) Sampling of a white colony with an autoclaved bamboo skewer. (**G**) Inoculation of a sampled colony into LB liquid medium containing antibiotics. (**H**) Placement of tubes with the liquid medium in a shaking incubator for growth.

3 To construct a TALE array that recognizes the designed sequence, select the pFUS2 vector and the appropriate modules from the Platinum Gate TALEN Kit.
  Refer to the Platinum Gate TALEN Kit protocol for guidance on this selection process.
  The first procedure for constructing a binary vector is to make intermediate plasmids based on the protocol for assembly ‐Step 1‐ of the Platinum Gate TALEN Kit. In steps 3 to 15, we introduce this protocol with slight modifications tailored to our specific requirements.
4 Prepare a master mix of distilled water, enzymes, and buffer for the Golden Gate reaction (see Table 3).
5 To perform the Golden Gate reaction, prepare the reaction mixture in an 8‐strip 0.2‐ml PCR tube following the specifications outlined in Table 3. Place the tube in a thermal cycler and run the following program: 5 min at 37°C, followed by 10 min at 16°C, repeating this cycle for a total of six times.
6 To eliminate any remaining material plasmids, add 0.25 µl rCutSmart^™^ Buffer and 0.1 µl BsaI‐HFv2 to the reaction solution from the previous step. Incubate at 37°C for 1 hr, followed by heat inactivation at 80°C for 5 min.
  Prepare a master mix of the enzymes and the buffer in advance.
7 Dispense 25 µl Competent Quick DH5α into a 1.5‐ml tube and then add 2 µl of the reaction solution. Subsequently, place the tube on ice for 30 min (Fig. 3A).

8 Incubate the 1.5‐ml tube in a 1.5‐ml tube incubator at 42°C for 45 s (Fig. 3B).
9 After the heat shock, place the tube on ice for 1 min. Then, add 250 µl SOC medium (Fig. 3C) and incubate the mixture at 37°C for 1 hr in the 1.5‐ml tube incubator (Fig. 3D).
10 Prepare a mixture of 50 µl each of 0.1 M IPTG solution and 20 mg ml^−1^ X‐Gal solution. Spread this mixture on an LB spectinomycin plate using a bacteria spreader.
  Prepare a master mix of IPTG and X‐Gal in advance.
11 Spread 100 µl of the *E. coli* culture onto the plate using a bacteria spreader (Fig. 3E). Incubate the plate at 37°C overnight in a bacterial incubator.
12 Aliquot 2 ml LB spectinomycin medium into a 14‐ml culture tube.
13 Using an autoclaved bamboo skewer or similar implement, pick a white colony from the plate (see step 11) and transfer it into the liquid medium (Fig. 3F and 3G). Remove the skewer and incubate the tube overnight at 37°C with shaking at 200 rpm using a shaking incubator (Fig. 3H).
14 Extract plasmids from the *E. coli* culture using a plasmid extraction kit (including centrifugation). Measure the plasmid concentration with a spectrophotometer and perform Sanger sequencing using pCR8‐F1 as the sequencing primer.
  We outsource Sanger sequencing to Eurofins Genomics.
15 Verify the sequencing data using sequence analysis software to confirm successful acquisition of the target plasmid.

#### Assembly Step 2

16 Dilute the plasmids prepared in Assembly Step 1 (pFUS2_a and pFUS2_b; see step 14) and the E1 and E3 vectors to a final concentration of 50 ng/µl using TE (pH 8.0).
  Following the assembly ‐Step 2‐ protocol of the Platinum Gate TALEN Kit, intermediate plasmids encoding the left and right ORFs of TALECD are constructed. Because our method differs slightly from the kit protocol, steps 16 to 30 include these variations. Instead of the ptCMV vector provided in the kit, we use plasmids starting with E1 or E3, as listed in Table 1.
17 Prepare a master mix of distilled water, enzymes, and buffer for the Golden Gate reaction (see Table 4).
18 To perform the Golden Gate reaction, prepare the reaction mixture in an 8‐strip 0.2‐ml PCR tube following the specifications outlined in Table 4. Place the tube in a thermal cycler and run the following program: 5 min at 37°C, followed by 10 min at 16°C, repeated for 10 cycles.
19 Perform an additional digestion to eliminate undigested material plasmids by adding 0.5 µl Tango Buffer (10×), 0.5 µl of 10 mM dithiothreitol, and 0.2 µl BsmBI to the reaction mixture. Incubate at 37°C for 1 hr, followed by heat inactivation at 80°C for 5 min.
  Prepare a master mix of the enzyme, dithiothreitol, and the buffer in advance.
20 Dispense 25 µl Competent Quick DH5α into a 1.5‐ml tube and then add 2 µl of the reaction solution. Subsequently, place the tube on ice for 30 min.
21 Incubate the 1.5‐ml tube in a 1.5‐ml tube incubator at 42°C for 45 s. Immediately transfer the tube back to ice for 1 min before adding 250 µl SOC medium, followed by incubation at 37°C for 1 hr.
22 Spread a mixture of 50 µl of 0.1 M IPTG and 50 µl of 20 mg ml^−1^ X‐Gal solution onto an LB kanamycin plate using a bacteria spreader.
  Prepare an IPTG and X‐Gal master mix in advance.
23 Spread 200 µl of the *E. coli* culture onto the prepared LB plate using a bacteria spreader. Incubate the plate at 37°C overnight.
24 To confirm the presence of the desired plasmid, conduct colony PCR. In an 8‐strip 0.2‐ml PCR tube, mix 4.4 µl sterile distilled water, 0.3 µl each of TAL‐F1 and TAL‐R2 primers, and 5.0 µl KOD One^®^ PCR Master Mix ‐Blue‐. Using a sterile pipet tip, select a white colony, briefly mix it into the PCR mixture, and then remove the tip. Perform amplification using a thermal cycler under the following conditions:

**Table:** 

Initial step:	30 s	94°C
25 cycles:	10 s	98°C
	5 s	60°C
	12 s	68°C.
  Prepare a master mix of the PCR reaction components in advance.
25 Load 1 µl of the PCR reaction product, alongside 250 bp DNA Ladder (Dye Plus), onto a 1% agarose gel and run electrophoresis at 100 V for 35 min in the Mupid‐2plus system filled with 1× TBE buffer.
26 Document the gel image using the Gel Doc EZ System with a compatible PC to confirm the presence of the desired amplicon.
  Expected band lengths corresponding to the number of TAL repeats are provided in Table 5.

**Table 5:** Length of PCR Products Corresponding to the Number of TAL Repeats

PCR product (bp)	Number of TAL repeats
824	6
926	7
1028	8
1130	9
1232	10
1334	11
1436	12
1538	13
1640	14
1742	15
1844	16
1946	17
2048	18
2150	19
2252	20
2354	21

27 Dispense 2 ml LB kanamycin medium into a 14‐ml culture tube.
28 Using an autoclaved bamboo skewer or similar implement, transfer the selected colony from the colony PCR (see step 24) to the liquid medium. After discarding the skewer, incubate the culture at 37°C and 200 rpm overnight using a shaking incubator.
29 Extract plasmids using a plasmid extraction kit and measure the plasmid concentration using a spectrophotometer. Then, perform Sanger sequencing using TAL‐F2 and TAL‐R2 primers.
  We outsource Sanger sequencing to Eurofins Genomics.
30 Analyze the sequencing data using sequence analysis software to confirm the presence of the desired plasmid sequence.

#### Assembly Step 3

31 Dilute the E1 and E3 vectors prepared in Assembly Step 2 (see step 29), as well as the E2_pENTR_R4R3_T CTP vector, to a concentration of 25 ng/µl using TE (pH 8.0).
  In steps 31 to 47, a binary vector is constructed to tandemly express a pair of either ptpTALECD or ptpTALECD_v2.
32 To initiate the LR reaction, mix 2.5 fmol of each diluted vector from step 31 and 5.0 fmol of the pDest_pK7WG2_pRPS5A_CTP_OleGFP vector in an 8‐strip 0.2‐ml PCR tube, adjusting the final volume to 2 µl with TE (pH 8.0). Add 0.5 µl LR Clonase™ II Plus enzyme mix and incubate at 37°C for 16 hr.
  If no colonies containing the desired plasmid are obtained, extending the incubation time to ≥40 hr may improve efficiency.
33 Following the incubation, add 0.5 µl proteinase K to the reaction and incubate at 37°C for 10 min to degrade remaining enzymes.
34 Dispense 25 µl *E. coli* HST08 Premium Competent Cells into a 1.5‐ml tube and then add 2 µl of the reaction mixture. Incubate the cells on ice for 30 min.
35 Heat‐shock the cells by placing the tube in a 1.5‐ml tube incubator set at 42°C for 45 s. Immediately following the heat shock, add 250 µl SOC medium without returning the tube to ice and incubate the culture at 37°C for 1 hr.
36 Spread the entire culture onto an LB spectinomycin plate using a bacteria spreader. Incubate the plate at 37°C overnight.
37 To confirm successful plasmid integration, perform colony PCR. In an 8‐strip 0.2‐ml PCR tube, mix 4.4 µl sterile distilled water, 0.3 µl each of the pK7WG2_fsFw and tHSP_fsRv primers, and 5.0 µl KOD One^®^ PCR Master Mix ‐Blue‐. Using a pipet tip, pick a single colony, resuspend it in the PCR mix, and discard the tip. Amplify the target sequence in a thermal cycler under the following conditions:

**Table:** 

Initial step:	30 s	94°C
25 cycles:	10 s	98°C
	5 s	55°C
	30 s	68°C.
  Prepare a master mix of the PCR reaction components in advance.
38 Load 1 µl of the PCR product, along with 1 kb DNA Ladder (Dye Plus), into the wells of a 1% agarose gel. Perform electrophoresis at 100 V for 45 min in the Mupid‐2plus system filled with 1× TBE buffer.
39 Capture an image of the gel using Gel Doc EZ System and verify the presence of a band of the expected size (5 to 6 kb).
40 Dispense 2 ml LB spectinomycin medium into a 14‐ml culture tube.
41 Using an autoclaved bamboo skewer or similar implement, transfer the colony confirmed by PCR (see step 37) into the liquid medium. After discarding the skewer, incubate the culture at 37°C and 200 rpm overnight in a shaking incubator.
42 Extract the plasmids from the *E. coli* culture using a plasmid extraction kit and measure the plasmid concentration using a spectrophotometer.
43 To further confirm the presence of the desired plasmid, perform a restriction enzyme digest according to the components listed in Table 6. Incubate the reaction at 37°C for 2 hr.
  The specific restriction enzymes used will depend on the position of the CD split and whether the C‐terminal side is fused to TAL Left or TAL Right, as indicated in Table 6.

**Table 6:** Composition of the Reaction Mixture for Restriction Enzyme Digestion of Binary Vectors

TALE Left‐Right; 1333C‐1333N or 1397C‐1397N		TALE Left‐Right; 1333N‐1333C		TALE Left‐Right; 1397N‐1397C	
Plasmid	x µl (100 ng)	Plasmid	x µl (100 ng)	Plasmid	x µl (100 ng)
rCutSmart^TM^ Buffer (New England Biolabs, cat. no. B6004S)	1 µl	rCutSmart^TM^ Buffer (New England Biolabs, cat. no. B6004S)	1 µl	rCutSmart^TM^ Buffer (New England Biolabs, cat. no. B6004S)	1 µl
Restriction enzyme 1	0.2 µl	BstEII‐HF (New England Biolabs, cat. no. R3162L)	0.2 µl	AscI (New England Biolabs, cat. no. R0558S)	0.2 µl
Restriction enzyme 2	0.2 µl	XhoI (New England Biolabs, cat. no. R0146L)	0.2 µl	EcoRI‐HF (New England Biolabs, cat. no. R3101L)	0.2 µl
Sterile distilled water (e.g., Nippon Gene, cat. no. 312‐90103)	8.6‐x µl	Sterile distilled water (e.g., Nippon Gene, cat. no. 312‐90103)	8.6‐x µl	BstEII‐HF (New England Biolabs, cat. no. R3162L)	0.2 µl
Total	10 µl	Total	10 µl	Sterile distilled water (e.g., Nippon Gene, cat. no. 312‐90103)	8.4‐x µl
				Total	10 µl

44 Following the digest, add 2 µl Gel Loading Dye, Purple (6×), to the reaction mixture and load 3 µl of the digested product alongside 3 µl of 1 kb DNA Ladder (Dye Plus) onto a 1% agarose gel. Perform electrophoresis at 100 V for 45 min in the Mupid‐2plus system filled with 1× TBE buffer.
45 Document the gel image using the Gel Doc EZ System and verify the presence of bands of the expected size.
  Band patterns can be predicted in advance using sequence analysis software such as Geneious Prime.
46 Conduct Sanger sequencing using four distinct sequencing primers for binary vectors.
  The sequencing primers are listed in Table 2 and may vary depending on the position of the CD split and whether the construct is ptpTALECD or ptpTALECD_v2.
  We outsource Sanger sequencing to Eurofins Genomics.
47 Analyze the sequencing data using sequence analysis software and confirm the presence of the desired plasmid. If correct, proceed to Basic Protocol 2.

## Basic Protocol 2 *Agrobacterium*‐MEDIATED INTRODUCTION OF A BINARY VECTOR INTO THE *Arabidopsis* NUCLEAR GENOME

This protocol describes the methods for cultivating Col‐0 and conducting floral dipping. Col‐0 plants are grown under long‐day conditions to promote rapid growth. Although the floral dip technique is based on an established protocol (Clough & Bent, 1998), it includes slight modifications that are detailed within the present protocol.

### Materials

Autoclaved ultrapure water
12% (w/v) sodium hypochlorite solution (Fujifilm Wako, cat. no. 197‐02206)
Tween‐20 (Sigma‐Aldrich, cat. no. P1379)
Seeds of *Arabidopsis thaliana* ecotype Col‐0
70% (v/v) ethanol (EtOH; see recipe)
1/2 MS agar plate (see recipe)
Binary vector (see Basic Protocol 1)
C58C1 Competent *Agrobacterium* (e.g., Filgen)
SOC medium (e.g., that included with *E. coli* HST08 Premium Competent Cells, TaKaRa, cat. no. 9128)
LB liquid medium (see recipe)
25 mg ml^−1^ gentamycin (see recipe)
LB spectinomycin plate (see recipe)
LB spectinomycin medium (see recipe)
100 mg ml^−1^ spectinomycin (see recipe)
50 mg ml^−1^ rifampicin (see recipe)
Sucrose (Fujifilm Wako, cat. no. 192‐00017)
SILWET L‐77 (BMS, cat. no. BMS‐SL7755)

1.5‐ml tubes (e.g., INA OPTICA, cat. no. CF‐0150)
Vortex mixer (e.g., Vortex Genie2, M&S Instruments)
Microcentrifuge
Vortex shaker (e.g., MicroMixer E‐36, TAITEC)
Laminar flow cabinet (e.g., VST‐1001, Nippon Medical & Chemical Instruments)
Surgical tape (e.g., 3M, cat. no. 1530‐0)
Aluminum foil
Plant growth chamber (e.g., Nippon Medical & Chemical Instruments)
Sterile tweezers
Fully hydrated, autoclaved Jiffy‐7 pellet (Jiffy‐7 44 mm, Jiffy Group International)
Scissors
NEPA cuvette electrode, 2‐mm gap (green) (NEPA Gene, cat. no. EC‐002)
Kimwipes
MicroPulser^TM^ Electroporator (Bio‐Rad, cat. no. 1652100)
Incubator (e.g., IC402, Yamato Scientific), 28°C
Bacteria spreader (e.g., AS ONE, cat. no. 1‐3192‐01)
14‐ml culture tubes (e.g., Corning Life Sciences, cat. no. 352059)
Autoclaved bamboo skewer or similar implement
Shaking incubator (e.g., Bioshaker, TAITEC, cat. no. BR‐43FL)
Plastic sheet (e.g., cut plastic bag)
Tray
50‐ml centrifuge tubes (e.g., WATSON, cat. no. 1342‐050S)
Analytical balance
Standard tabletop centrifuge (e.g., LC‐200, TOMY)
Magnetic stir bar
300‐ml disposable cup (e.g., AS ONE, cat. no. 5‐077‐04)
Magnetic stirrer
OHP film
Plastic wrap
Autoclave (e.g., TOMY)

### Cultivation of wild‐type plants of Arabidopsis thaliana

**Figure 4:**
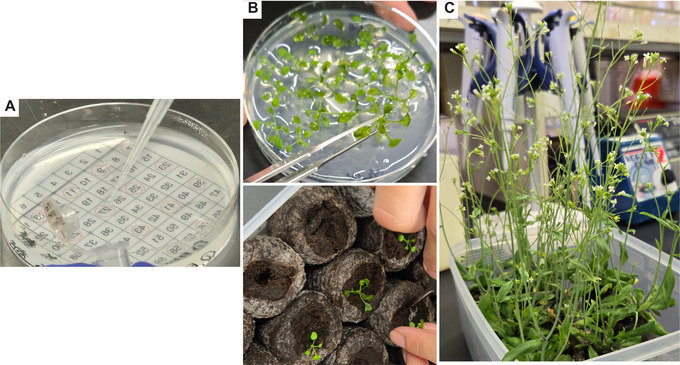
Cultivation of *Arabidopsis thaliana*. (**A**) Sowing of sterilized seeds on a 1/2 MS agar plate. (**B**) Transfer of seedlings (top) to autoclaved Jiffy‐7 pellets (bottom). (**C**) Plantlets subjected to floral dipping.

1 Prepare a seed sterilization solution consisting of 950 µl autoclaved ultrapure water, 50 µl of 12% sodium hypochlorite solution, and 5 µl Tween‐20 in a 1.5‐ml tube.
2 Transfer about 100 seeds of *Arabidopsis thaliana* ecotype Col‐0 into a 1.5‐ml tube and add 1 ml of 70% EtOH. Sterilize the seeds’ surface by vortexing the mixture for 30 s.
3 Centrifuge the tube for 30 s at 13,000 rpm and then carefully discard the 70% EtOH.
4 Add the sterilization solution prepared in step 1 to the tube, agitate for 15 min using a vortex shaker, and centrifuge briefly.
5 In a laminar flow cabinet, remove the sterilization solution and replace it with 1000 µl autoclaved ultrapure water. Briefly vortex and centrifuge the tube and then discard the liquid. Repeat this rinsing process four times total to ensure thorough washing.
  Conduct steps 5 to 7 (until storage) in a laminar flow cabinet.
6 Add a final 1000 µl of autoclaved ultrapure water to the tube. Using a 1000‐µl pipet, carefully sow each seed onto a 1/2 MS agar plate (Fig. 4A).

7 Seal the edges of the plate with surgical tape, wrap it in aluminum foil to prevent light exposure, and store it at 4°C for 5 days to promote germination.
8 Transfer the plate to long‐day growth conditions (16 hr light/8 hr dark) at 22°C in a plant growth chamber and allow the seedlings to grow for 2 to 3 weeks. Once the seedlings have developed sufficiently, use sterile tweezers to transplant each one into a fully hydrated, autoclaved Jiffy‐7 pellet (Fig. 4B).
  Hereafter, plants are cultivated under the same conditions.
9 To promote the development of multiple flowering stalks, excise the first inflorescence with scissors. Approximately 2 to 4 weeks after transplantation, when each plant has developed three or more stalks (Fig. 4C), introduce the binary vector into the nuclear genome via the floral dip method (Clough and Bent, 1998) by proceeding to the next steps.

### Agrobacterium transformation

**Figure 5:**
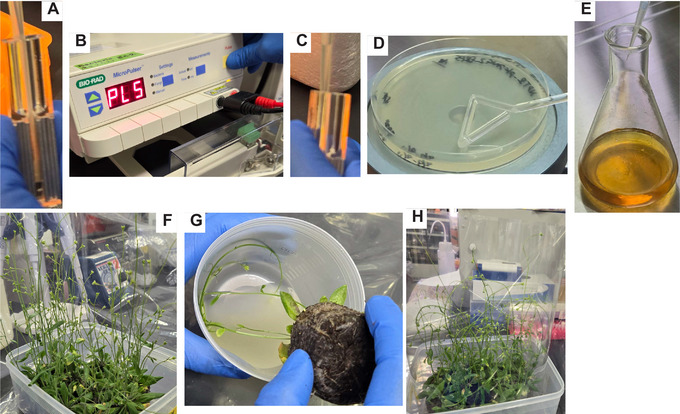
Procedures for transformation and culture of *Agrobacterium* and floral dipping. (**A**) Transfer of *Agrobacterium* cells mixed with a binary vector into an electroporation cuvette. (**B**) Application of an electrical pulse to facilitate vector uptake into the cells. (**C**) Addition of SOC medium to the cuvette following electroporation. (**D**) Plating of the bacterial suspension onto an LB solid plate containing spectinomycin after 1 to 3 hr of incubation at 28°C. (**E**) Addition of the bacterial suspension cultured in a 14‐ml tube to LB liquid medium supplemented with spectinomycin, rifampicin, and gentamicin. (**F**) Excision of flowers and siliques from the plant. (**G**) Immersion of flower buds in the *Agrobacterium* suspension, prepared by centrifuging the bacterial culture, discarding the supernatant, and resuspending the pellet in a sucrose solution. (**H**) Covering the plants with OHP film after floral dipping to maintain humidity.

10 Add 0.2 µl of the binary vector to 50 µl C58C1 Competent *Agrobacterium* (e.g., Filgen) and transfer the mixture to a NEPA cuvette electrode, 2‐mm gap (green) (Fig. 5A). Place the cuvette on ice for 1 min.

11 Carefully remove any water droplets from the electrode using a Kimwipe and insert the cuvette into the MicroPulser^TM^ Electroporator. Apply an electric pulse with the internal program Ec2, set at 2.5 kV for a few seconds (Fig. 5B).
12 Add 200 µl SOC medium to the cuvette using a 200‐µl pipet (Fig. 5C) and pipet the mixture up and down gently several times. Subsequently, transfer the mixture to a new 1.5‐ml tube and incubate the tube in an incubator at 28°C for 1 to 3 hr to allow bacterial recovery.
13 While incubating the bacteria, mix 100 µl LB liquid medium with 20 µl of 25 mg ml^−1^ gentamycin in a laminar flow cabinet. Spread this mixture onto an LB spectinomycin plate using a bacteria spreader.
  Steps 13 and 14 should both be performed in a laminar flow cabinet.
14 Evenly spread the bacterial solution onto the plate (Fig. 5D) and seal the edges with surgical tape.
15 Wrap the plate in aluminum foil and incubate at 28°C for 2 days.
16 Two days before floral dipping (steps 19 to 27), dispense 2 ml LB spectinomycin medium into a  4‐ml culture tube in a laminar flow cabinet.
17 In the laminar flow cabinet, use an autoclaved bamboo skewer or similar implement to select a colony from the plate from step 15 and transfer it into the liquid medium. After discarding the bamboo skewer, incubate the tube overnight at 28°C and 130 rpm using a shaking incubator.
18 Prepare a larger‐scale culture by adding 100 µl of 100 mg ml^−1^ spectinomycin, 50 µl of 50 mg ml^−1^ rifampicin, and 25 µl of 25 mg ml^−1^ gentamycin to 100 ml LB liquid medium. Then, add 200 µl of the overnight bacterial culture and shake the mixture at 28°C and 130 rpm for ∼24 hr using a shaking incubator (Fig. 5E).

### Floral dipping

19 To prevent contamination during the floral dip process, cover the workspace with a plastic sheet (e.g., a cut plastic bag).
20 Prior to the dip, trim off any open flowers and siliques from the plants using scissors (Fig. 5F) and place them on a tray.
  Use 8 to 15 plants for transformation.
21 Divide the bacterial solution equally between two 50‐ml centrifuge tubes using an analytical balance and centrifuge 15 min at 1580 × *g*.
22 During centrifugation, prepare the bacterial suspension solution by placing a magnetic stir bar into a 300‐ml disposable cup on a magnetic stirrer. Add 75 ml autoclaved ultrapure water and 7.5 g sucrose, stirring until dissolved. Then, add 37.5 µl SILWET L‐77 and continue stirring.
23 After centrifugation (step 21), discard the supernatant and resuspend the bacterial pellet in 5 ml of the prepared solution using a vortex mixer. Add the remaining solution, mix by gentle inversion, and transfer the full volume into the 300‐ml disposable cup.
24 Dip the flower buds from step 20 into the bacterial suspension for 20 to 30 s (Fig. 5G), repeating the process once after a short interval. Once all plants have been treated, return them to the tray, enclose them with OHP film, and cover with plastic wrap (Fig. 5H).
25 Autoclave all materials that came into contact with the bacterial solution to prevent contamination.
26 Remove the plastic wrap the following day.
27 Approximately 1 month after floral dipping, harvest the seeds and proceed to screening for transgenic lines (see Basic Protocol 3).

## Basic Protocol 3 SELECTION OF PLANTS HARBORING T‐DNA IN THE NUCLEUS AND DETECTION OF BASE EDITING IN THE PLASTID GENOME

This protocol outlines the method to evaluate whether the targeted bases in the plastid genome of the generated transformants (Basic Protocol 2) are successfully substituted. Typically, the targeted bases are homoplasmically substituted in the T_1_ generation, with the substitutions stably inherited by the T_2_ generation. Some of these T_2_ plants lack the foreign gene introduced into the nuclear genome and thus are so‐called null segregants. As no further base substitutions are expected in these null segregants and their progeny, we usually use the next‐generation T_3_ plants for phenotypic analysis.

### Materials

Seeds harvested in Basic Protocol 2
*Arabidopsis thaliana* ecotype Col‐0 seeds
1/2 MS agar plate containing 125 mg L^−1^ Claforan (see recipe)
Buffer for roughly extracting DNA (see recipe)
250 bp DNA Ladder (Dye Plus) (TaKaRa, cat. no. 3424A)
1% (w/v) agarose gel
1× TBE buffer (see recipe)
Gel/PCR extraction kit (e.g., Nippon Genetics, cat. no. FG‐91302)
Liquid nitrogen
Maxwell^®^ RSC Plant DNA Kit (Promega, cat. no. AS1490)

35‐mm‐diameter dish (e.g., IWAKI, cat. no. 1000‐035)
Fluorescence stereomicroscope
Seed picker (EASE‐UP, cat. no. BC‐SDP01)
1.5‐ml tubes (e.g., INA OPTICA, cat. no. CF‐0150)
Plant growth chamber (e.g., Nippon Medical & Chemical Instruments)
Laminar flow cabinet
8‐strip 0.2‐ml PCR tubes (e.g., Nippon Genetics, cat. no. FG‐028DC)
Laminar flow cabinet (e.g., VST‐1001, Nippon Medical & Chemical Instruments)
Sterile scissors
Thermal cycler (e.g., Applied Biosystems^TM^ MiniAmp Plus, Thermo Fisher Scientific)
Mupid‐2plus system (TaKaRa)
Gel Doc EZ System (Bio‐Rad) with compatible PC
Spectrophotometer (NanoDrop One^C^, Thermo Fisher Scientific)
Sequence analysis software (e.g., Geneious Prime)
Fully hydrated, autoclaved Jiffy‐7 pellet (Jiffy‐7 44 mm, Jiffy Group International)
Sterile tweezers
Plant growth chamber (e.g., Nippon Medical & Chemical Instruments)
2.0‐ml crushing tube (2.0‐ml Master Tube Hard, BMS, cat. no. MT020‐01H)
Zirconia Ball φ5mm (NIKKATO, cat. no. 5‐4060‐33)
Multi‐beads Shocker (Yasui Kikai)
Maxwell^®^ RSC Instrument (Promega) with compatible computer

Additional reagents and equipment for aseptically sowing seeds (see Basic Protocol 2, steps 1 to 7), preparing PCR mixture (see Table 7), running agarose gel electrophoresis (see Current Protocols article: Voytas, 2000), and performing Sanger sequencing and next‐generation sequencing (NGS)

**Table 7:** Composition of the PCR Mixture to Amplify the Base Editor Gene or the Target Window

Target: Base editor gene		Target: Target window	
Sterile distilled water (e.g., Nippon Gene, cat. no. 312‐90103)	4.1 µl	Sterile distilled water (e.g., Nippon Gene, cat. no. 312‐90103)	3.9 µl
CTP_Fw (5′‐ ATGGATTCACAGCTAGTCTTGTCTC ‐3′)	0.2 µl	Fw primer corresponding to a target region	0.3 µl
TAL_F1r (5′‐ CCACTGTTTGCCGACGCCAA ‐3′)	0.2 µl	Rv primer corresponding to a target region	0.3 µl
Quick Taq^®^ HS DyeMix (TOYOBO, cat. no. DTM‐101)	5.0 µl	KOD One^®^ PCR Master Mix ‐Blue‐ (TOYOBO, cat. no. KMM‐201)	5.0 µl
Crude extract	0.5 µl	Crude extract	0.5 µl
Total	10.0 µl	Total	10.0 µl

### Genotyping of T_1_ plants

1 Spread the seeds harvested in Basic Protocol 2 onto a 35‐mm‐diameter dish.
2 Observe the seeds under a fluorescence stereomicroscope, exposing them to green fluorescent protein (GFP) excitation light. Use a seed picker to transfer seeds exhibiting GFP fluorescence to a 1.5‐ml tube.
  GFP serves as a selection marker expressed by the transgene.
3 Aseptically sow the selected seeds, along with *Arabidopsis thaliana* ecotype Col‐0 seeds, onto a 1/2 MS agar plate containing 125 mg L⁻¹ Claforan according to steps 1 to 7 of Basic Protocol 2.
4 Following cold treatment, grow the seedlings for ∼10 days under long‐day conditions (16 hr light/8 hr dark) at 22°C in a plant growth chamber.
5 Cut a cotyledon or a true leaf within a laminar flow cabinet using sterile scissors and place cotyledon or leaf in an 8‐strip 0.2‐ml PCR tube pre‐filled with 50 µl of buffer for roughly extracting DNA.
  To prevent cross‐contamination, sterilize the scissors by dipping them in 70% (v/v) EtOH (see recipe) and flame‐sterilizing with a gas burner before cutting each sample.
6 Set the 8‐strip tube in a thermal cycler and incubate at 98°C for 15 min.
7 To detect the presence of the base editor gene, conduct PCR in a 0.2‐ml PCR tube. Prepare the PCR mixture according to the composition specified in Table 7, place the tube in a thermal cycler, and then perform amplification under the following conditions:

**Table:** 

Initial step:	2 min	94°C
38 cycles:	30 s	94°C
	30 s	60°C
	30 s	68°C.
  Prepare a master mix of the PCR reaction in advance. Use the Col‐0 crude extract and water as the negative‐control template.
8 Load 1 µl of the PCR reaction mixture alongside 250 bp DNA Ladder (Dye Plus) into the wells of a 1% agarose gel and perform electrophoresis at 100 V for 35 min in the Mupid‐2plus system filled with 1× TBE buffer.
9 Capture an image of the gel using the Gel Doc EZ System with a compatible PC and verify the presence of the expected 480‐bp band.
10 For samples in which the base editor gene is detected, perform PCR to amplify the target sequence. Prepare the PCR mixture according to the composition specified in Table 7 and run amplification in a thermal cycler under the following conditions:

**Table:** 

Initial step:	30 s	94°C
35 cycles:	10 s	98°C
	5 s	X°C
	Y s	68°C.
  X and Y are dependent on the Tm values of the primers and the length of the PCR product, respectively. Typically, X is set to 2°C below the lower Tm value of the two primers, and Y is adjusted according to the KOD One^®^ PCR Master Mix ‐Blue‐ protocol.
  Prepare the PCR master mix in advance and use water as negative control.
11 Load 1 µl of the PCR reaction mixture alongside 2 µl of 250 bp DNA Ladder (Dye Plus) into a 1% agarose gel and perform electrophoresis at 100 V for 35 min in 1× TBE Buffer using the Mupid‐2plus system filled with 1× TBE buffer.
12 After electrophoresis, capture a gel image using the Gel Doc EZ System to confirm the presence of the expected target band.
13 Purify the PCR product using a Gel/PCR extraction kit and measure its concentration using a spectrophotometer.
14 Perform Sanger sequencing with primers that anneal 150 to 300 bp distant from the target base.
  We outsource sequencing to Eurofins Genomics.
15 Analyze the sequencing data with sequence analysis software to verify the presence of the desired base substitution (Fig. 6).

**Figure 6:**
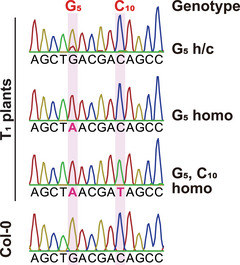
Examples of Sanger traces of T_1_ plants and Col‐0. Sanger traces of a target window within *16S rRNA* are shown. G_5_ and C_10_ indicate the 5^th^ G and 10^th^ C in the target window, respectively. Homo, homoplasmically substituted; h/c, heteroplasmically or chimerically substituted. Modified from Nakazato et al. (2021).

16 Transfer each plant with the desired base substitution to a fully hydrated, autoclaved Jiffy‐7 pellet using sterile tweezers and cultivate it under long‐day growth conditions (16 hr light/8 hr dark) at 22°C in a plant growth chamber.

### Genotyping of T_2_ plants

17 Harvest seeds from the T_1_ plants approximately 1 to 2 months after transfer to soil. Subsequently, select seeds exhibiting no GFP fluorescence in accordance with the procedures described in steps 1 and 2.
18 Sow the selected seeds onto a 1/2 MS agar plate, as in steps 1 to 7 of Basic Protocol 2.
19 To confirm the absence of the base editor gene, conduct sampling of a leaf, PCR amplification, and electrophoresis as described in steps 5 to 9.
  For the negative‐control template in PCR, use sterile distilled water and crude extracts from Col‐0. In addition, use crude extracts from a T_1_ plant as the positive control.
20 In samples where the base editor gene is not detected, investigate the inheritance of the substituted base(s). Conduct amplification of the target window by PCR, electrophoresis, purification of the PCR products, Sanger sequencing, and subsequent analysis as detailed in steps 10 to 15.
21 Transplant the plants that are confirmed to have inherited the substituted base(s) using the methodology described in step 16. Harvest the seeds and utilize them for phenotypic analysis (e.g., Crushi measuring photosynthetic activity or biomass).
  Identifying plants that do not possess the base editor gene in their genome (so‐called null segregants) while inheriting substituted base(s) minimizes the likelihood of additional base substitutions, thereby facilitating a clearer analysis of the relationship between genotype and phenotype.
22 If necessary, examine the presence of off‐target mutations in the plastid genome of T_2_ plants that have inherited the substituted base(s) and do not have the base editor gene. To do so, excise a leaf with sterilized scissors, place it in a 2.0‐ml crushing tube containing a Zirconia Ball φ5mm, and then immediately freeze the tube in liquid nitrogen.
  To prevent cross‐contamination between samples, scissors should be immersed in 70% (v/v) EtOH (see recipe) and wiped with a paper towel prior to cutting leaves from different individuals.
23 Subject the tube to freeze‐grinding at 1800 rpm for 30 s using a Multi‐beads Shocker, followed by total DNA extraction using the Maxwell^®^ RSC Plant DNA Kit in conjunction with the Maxwell^®^ RSC Instrument with a compatible computer.
24 Measure the concentration of total DNA using a spectrophotometer.
25 Outsource NGS to a company (e.g., Macrogen) and analyze the resultant data in accordance with the protocols established in our previous research (Nakazato et al., 2021) to investigate the potential presence of off‐target mutations.

## REAGENTS AND SOLUTIONS

### 1/2 MS agar plate

Dissolve 2.3 g Murashige and Skoog Plant Salt Mixture (Fujifilm Wako, cat. no. 392‐00591), 10 g sucrose (Fujifilm Wako, cat. no. 192‐00017), 500 mg of 2‐morpholinoethanesulfonic acid, monohydrate (Fujifilm Wako, cat. no. 345‐01625), 1 ml Plant Preservative Mixture (Plant Cell Technology), and 1 ml of 1000× MS vitamin (see recipe) into ∼900 ml ultrapure water in a 1000‐ml beaker with a stir bar. Adjust pH to 5.9 by adding 10 N KOH and using a pH meter (e.g., Horiba) and then bring the solution to a final volume of 1000 ml by using a 1000‐ml graduated cylinder. Subsequently, add 7 g agar (Fujifilm Wako, cat. no. 010‐15815) to the medium and autoclave at 121°C for 15 min under 2 atm. Stir the solution to dissolve the agar and then pour the mixture into 90‐mm‐diameter petri dishes (e.g., AS ONE, cat. no. 3‐1491‐01) in a laminar flow cabinet. Store ≤6 months at 4°C.

### 1/2 MS agar plate containing 125 mg L^−1^ Claforan

Dissolve 2.3 g Murashige and Skoog Plant Salt Mixture (Fujifilm Wako, cat. no. 392‐00591), 10 g sucrose (Fujifilm Wako, cat. no. 192‐00017), 500 mg of 2‐morpholinoethanesulfonic acid, monohydrate (Fujifilm Wako, cat. no. 345‐01625), 1 ml Plant Preservative Mixture (Plant Cell Technology), and 1 ml of 1000× MS vitamin (see recipe) into ∼900 ml ultrapure water in a 1000‐ml beaker with a stir bar. Adjust pH to 5.9 by adding 10 N KOH and using a pH meter (e.g., Horiba) and then bring the solution to a final volume of 1000 ml by using a 1000‐ml graduated cylinder. Subsequently, add 7 g agar (Fujifilm Wako, cat. no. 010‐15815) to the medium and autoclave at 121°C for 15 min under 2 atm. Stir the solution to dissolve the agar and then add 500 µl of 250 mg ml^−1^ Claforan (see recipe). Pour the mixture into 90‐mm‐diameter petri dishes (e.g., AS ONE, cat. no. 3‐1491‐01) in a laminar flow cabinet. Store ≤6 months at 4°C.

### Agarose gel, 1%

Add 1.5 g Agarose S (Nippon Gene, cat. no. 313‐90231) and a stir bar to 150 ml of 1× TBE Buffer (see recipe) in a 300‐ml Erlenmeyer flask. Heat the mixture in a microwave until the agarose is fully dissolved. Subsequently, introduce 150 µl of a 0.5 mg ml^−1^ ethidium bromide (EtBr) solution [prepared by diluting EtBr Solution (Nippon Gene, cat. no. 315‐90051) 20‐fold with ultrapure water] into the flask. Pour the resultant mixture into the gel maker set included with the Mupid‐2plus system (TaKaRa). Store ≤3 months at 4°C.

### Buffer for roughly extracting DNA

Dilute 1 M Tris·HCl, pH 9.5 (iNtRON Biotechnology, cat. no. IBS‐BT091), and 0.5 M EDTA (pH 8.0) (Nippon Gene, cat. no. 311‐90075) with ultrapure water to achieve a buffer with final concentrations of 100 mM and 10 mM, respectively. Store ≤6 months at 4°C.

### Claforan, 250 mg ml^−1^

Dissolve 1 g Claforan (Sanofi) in 4 ml ultrapure water and sterilize the solution using a Terumo Syringe [Terumo Syringe 10 ml for Vaccination Slip Tip (Side Entrance) White, TERUMO, cat. no. SS‐10ESZ] and Millex‐LG, 0.20 µm (Merck, cat. no. SLLG025SS) in a laminar flow cabinet. Store ≤1 year at –20°C.

### Dithiothreitol, 10 mM

Dilute 1 mol/L (±)‐Dithiothreitol (DTT) Solution (Fujifilm Wako, cat. no. 044‐33871) with sterilized ultrapure water. Store ≤6 months at –20°C.

### Ethanol (EtOH), 70%

Dilute 700 ml Ethanol (99.5) (Fujifilm Wako, cat. no. 057‐00451) with 300 ml ultrapure water. Store ≤6 months at –20°C.

### Gentamycin, 25 mg ml^−1^

Dissolve 3.01 g gentamicin sulfate (Fujifilm Wako, cat. no. 079‐02974) in 100 ml ultrapure water and sterilize the solution using a Terumo Syringe [Terumo Syringe 10 ml for Vaccination Slip Tip (Side Entrance) White, TERUMO, cat. no. SS‐10ESZ] and Millex‐LG, 0.20 µm (Merck, cat. no. SLLG025SS) in a laminar flow cabinet. Store ≤1 year at –20°C.

### IPTG solution, 0.1 M

Dissolve 0.238 g IPTG (dioxane free) (Isopropyl‐β‐d‐thiogalactopyranoside) (TaKaRa, cat. no. 9030) in 10 ml ultrapure water. Then, sterilize this solution with a Terumo Syringe [Terumo Syringe 10 ml for Vaccination Slip Tip (Side Entrance) White, TERUMO, cat. no. SS‐10ESZ] and Millex‐LG, 0.20 µm (Merck, cat. no. SLLG025SS) in a laminar flow cabinet. Store ≤6 months at –20°C.

### Kanamycin, 50 mg ml^−1^

Dissolve 0.5 g kanamycin sulfate (Fujifim Wako, cat. no. 113‐00341) in 10 ml ultrapure water and sterilize the solution using a Terumo Syringe [Terumo Syringe 10 ml for Vaccination Slip Tip (Side Entrance) White, TERUMO, cat. no. SS‐10ESZ] and Millex‐LG, 0.20 µm (Merck, cat. no. SLLG025SS) in a laminar flow cabinet. Store ≤6 months at –20°C.

### LB kanamycin medium

Autoclave LB medium (see recipe) under 121°C for 15 min under 2 atm and then add 1000 µl of 50 mg ml^−1^ kanamycin (see recipe) in a laminar flow cabinet. Store ≤2 months at 4°C.

### LB kanamycin plate

Add 15 g agar (Fujifilm Wako, cat. no. 010‐15815) to 1 L LB medium (see recipe) and autoclave at 121°C for 15 min under 2 atm. Stir the solution to dissolve the agar and then add 1000 µl of 50 mg ml^−1^ kanamycin (see recipe). Pour the mixture into 90‐mm‐diameter petri dishes (e.g., AS ONE, cat. no. 3‐1491‐01) in a laminar flow cabinet. Store ≤2 months at 4°C.

### LB liquid medium

Prepare 1 L LB medium (see recipe), aliquot 100 ml into each 200‐ml Erlenmeyer flask, and autoclave at 121°C for 15 min. Store ≤2 months at ambient temperature.

### LB medium

Dissolve 10 g sodium chloride (Fujifilm Wako, cat. no. 195‐01663), 10 g hypolypepton (Fujifilm Wako, cat. no. 392‐02115), and 5 g yeast extract powder (Oriental Yeast, cat. no. 42007000) into ∼900 ml ultrapure water in a 1000‐ml beaker with a stir bar. Adjust pH by adding 500 µl of 10 N NaOH and then bring the solution to a final volume of 1000 ml by using a 1000‐ml graduated cylinder. Store ≤6 months at 4°C.

### LB spectinomycin medium

Autoclave LB medium (see recipe) under 121°C for 15 min under 2 atm and then add 1000 µl of 100 mg ml^−1^ spectinomycin (see recipe) in a laminar flow cabinet. Store ≤2 months at 4°C.

### LB spectinomycin plate

Add 15 g agar (Fujifilm Wako, cat. no. 010‐15815) to 1 L LB medium (see recipe) and autoclave at 121°C for 15 min under 2 atm. Stir the solution to dissolve the agar and then add 1000 µl of 100 mg ml^−1^ spectinomycin (see recipe). Pour the mixture into 90‐mm‐diameter petri dishes (e.g., AS ONE, cat. no. 3‐1491‐01) in a laminar flow cabinet. Store ≤2 months at 4°C.

### MS vitamin, 1000×

Dissolve 100 g myo‐inositol (Fujifilm Wako, cat. no. 094‐00281), 2 g glycine (Fujifilm Wako, cat. no. 073‐00732), 500 mg nicotinic acid (Fujifilm Wako, cat. no. 142‐01232), 500 mg pyridoxine hydrochloride (Sigma‐Aldrich, cat. no. P9755), and 100 mg thiamine hydrochloride (Fujifilm Wako, cat. no. 201‐00852) into ∼900 ml ultrapure water in a 1000‐ml beaker with a stir bar. Bring the solution to a final volume of 1000 ml by using a 1000‐ml graduated cylinder. Store ≤1 year at –20°C.

### Rifampicin, 50 mg ml^−1^

Dissolve 5 g rifampicin (Fujifilm Wako, cat. no. 185‐01003) in 100 ml dimethyl sulfoxide (Fujifilm Wako, cat. no. 043‐07216) and sterilize the solution using a Terumo Syringe [Terumo Syringe 10 ml for Vaccination Slip Tip (Side Entrance) White, TERUMO, cat. no. SS‐10ESZ] and Millex‐LG, 0.20 µm (Merck, cat. no. SLLG025SS) in a laminar flow cabinet. Store ≤1 year at –20°C.

### Spectinomycin, 100 mg ml^−1^

Dissolve 1.49 g spectinomycin dihydrochloride pentahydrate (Fujifim Wako, cat. no. 195‐11531) in 10 ml ultrapure water and sterilize the solution using a Terumo Syringe [Terumo Syringe 10 ml for Vaccination Slip Tip (Side Entrance) White, TERUMO, cat. no. SS‐10ESZ] and Millex‐LG, 0.20 µm (Merck, cat. no. SLLG025SS) in a laminar flow cabinet. Store ≤1 year at –20°C.

### TBE buffer, 1×

Dissolve 484.4 g of 2‐amino‐2‐hydroxymethyl‐1,3‐propanediol (Fujifilm Wako, cat. no. 206‐07884), 247.2 g boric acid (Fujifilm Wako, cat. no. 023‐02194), and 29.6 g Ethylenediamine‐*N,N,N',N'*‐tetraacetic Acid Disodium Salt Dihydrate (2Na) (Fujifilm Wako, cat. no. 349‐01863) in 3 L ultrapure water. Subsequently, dilute this 10× TBE buffer 10‐fold with ultrapure water to obtain 1× TBE buffer. Store ≤6 months at ambient temperature.

### X‐Gal solution, 20 mg ml^−1^

Dissolve 200 mg X‐Gal (Free S, cat. no. 510‐620‐G005‐X‐Gal) into 10 ml *N,N‐*dimethylformamide (Fujifilm Wako, cat. no. 049‐02914). Store ≤6 months at –20°C.

## COMMENTARY

### Background Information

Plastid genome‐editing methods were first reported in 2021 (Nakazato et al., 2021; Kang et al., 2021). With these methods, targeted C:G pairs were substituted with T:A pairs. Nakazato et al. (2021) successfully modified the plastid genome of Col‐0 using ptpTALECD (introduced in this article), whereas Kang et al. (2021) employed a similar base‐editing enzyme, named chloroplast‐targeted DddA‐derived cytosine base editor, to modify the plastid genome in cultured cells of oilseed rape and lettuce. In the same year, Huang et al. achieved targeted DNA double‐strand breaks in the plastid genome of tobacco using plastid‐targeted TALENs (Huang et al., 2021). The following year, Mok et al. successfully substituted targeted A:T pairs with G:C pairs in the plastid genomes of cultured lettuce cells and Col‐0 plantlets utilizing a genome‐editing enzyme known as TALE‐linked deaminase (Mok et al., 2022). Moreover, eight additional studies have been published on the development and modification of C:G‐to‐T:A and A:T‐to‐G:C base‐editing enzymes for the plastid genome, reporting successful modifications in the plastid genomes of Col‐0 and rice (Li et al., 2021; Nakazato et al., 2023; Zhang and Boch, 2023; Hu et al., 2024; Mok et al., 2024; Wang et al., 2024; Zhang et al., 2024; Zhou et al., 2024). For details regarding these enzymes and their experimental results, please refer to our review (Nakazato and Arimura, 2024). These technologies have potential for advancing basic research on the plastid genome. In particular, TALENs may enhance our understanding of the repair mechanisms associated with plastid DNA double‐strand breaks. Base‐editing technology is expected to elucidate the regulatory mechanisms involving the plastid genome through the conversion of specific amino acids, gene disruption by introducing stop codons, and modification of the three‐dimensional structure of DNA and RNA and gene expression control regions. Furthermore, these editing technologies could serve as foundational techniques for utilizing the plastid genome for crop breeding. Recently, Mok et al. and Nakazato et al. successfully generated *A. thaliana* plantlets that were resistant to herbicides targeting the D1 protein encoded by the plastid genome; this was achieved through the substitution of specific amino acids in the D1 protein (Mok et al., 2024; Nakazato et al., 2024). Given that methods for editing the plastid genome can, in principle, be applied to crop species that are amenable to foreign gene introduction into the nuclear genome, the horizontal transfer of these amino acid substitutions may facilitate the development of herbicide‐resistant crops. Additionally, modifications of photosynthesis‐related genes may result in increased biomass and yield.

### Critical Parameters

We recommend that readers employ ptpTALECD to substitute TC and AC and employ ptpTALECD_v2 to substitute GC and CC.

#### Basic Protocol 1, Assembly Steps 1 and 2

If desired plasmids are not obtained, the use of fresh BsaI‐HFv2, BsmBI, Quick Ligase, and their corresponding reaction buffers may help resolve the issue.

#### Basic Protocol 1, step 32

If desired plasmids are not obtained, it is recommended to use fresh LR Clonase™ II Plus or to extend the reaction time to ≥40 hr.

#### Basic Protocol 1, steps 43 to 45

If incomplete plasmid digestion occurs, it is recommended to use fresh enzymes and to extend the reaction time to ensure optimal digestion.

#### Basic Protocol 3, step 2

If the number of GFP‐positive seeds is small, it is recommended to increase the number of Col‐0 plants subjected to floral dipping to enhance the likelihood of obtaining transformants.

#### Basic Protocol 3, step 15

If the target base is not substituted, but other bases several nucleotides away are modified, this issue may be addressed by designing constructs with the TALE recognition sequence shifted by several nucleotides and repeating the experiment. Additionally, when both the target base and adjacent bases are substituted, it is possible to isolate T_2_ null segregants in which only the target base is homoplasmically substituted while the other bases are wild type.

### Understanding Results

In Assembly Steps 1 and 2 in Basic Protocol 1, at least one of the two randomly selected white colonies will usually contain the desired plasmid. In Assembly Step 3, colonies harboring the desired plasmid are typically present at a ratio of 1 in 5 to 1 in 20. However, there may be instances where the number of colonies emerging ranges from a few to several dozen, yet none contains the desired plasmid. Several to approximately 100 T_1_ seeds are usually obtained per floral dip event. For each construct, individuals with substituted bases in the target window may account for 0% of 100% of the examined T_1_ individuals. Some T_1_ plants may have heteroplasmically or chimerically (i.e., not homoplasmically) substituted bases, but in T_2_ null segregants, nearly all of the bases will seem to be fixed to homoplasmy in either the wild‐type or edited state. When a homoplasmic substitution occurs exclusively at the target base in T_1_ plants, it is often observed that this homoplasmically substituted base is inherited by T_2_ null segregants, with no additional base edits. This pattern persists even when multiple bases are substituted in the T_1_ generation, as long as the homoplasmic substitution is restricted to the target base.

### Troubleshooting

See Table 8 for potential problems and their causes and solutions.

**Table 8:** Troubleshooting Guide for Targeted C‐to‐T Base Editing in the *Arabidopsis* Plastid Genome

Problem	Possible cause	Solution
Desired plasmids are not obtained	Enzymes are old	Use fresh enzymes
	Reaction efficiency is low (as for Assembly Step 3)	Incubate the reaction solution for ≥40 hr
Incomplete plasmid digestion	Restriction enzymes are old	Use fresh enzymes
Number of GFP‐positive T_1_ seeds is limited	Transformation efficiency is low	Subject more Col‐0 plants to floral dipping
Target base is not substituted	DddA could not access the target base	Design constructs with the TALE recognition sequence shifted by several nucleotides

### Time Considerations

#### Basic Protocol 1

Selection of TALE‐binding sequences: 1 hr

Plasmid construction: 3 weeks to 2 months

#### Basic Protocol 2

Cultivation of Col‐0: 1.5 months

Duration from *Agrobacterium*‐mediated transformation to the harvesting of T_1_ seeds: 5 weeks

#### Basic Protocol 3

Duration from selecting T_1_ seeds exhibiting GFP fluorescence to completing genotyping: 3 weeks

Duration until the collection of T_2_ seeds: 1.5 to 2 months

Duration from selecting T_2_ seeds not exhibiting GFP fluorescence to completing genotyping: 3 weeks

Extraction of total DNA: half a day

Duration until obtaining the results of NGS: 1 to 2 months

NGS analysis: 2 to 3 days

### Author Contributions

**Issei Naakazato**: Conceptualization; data curation; investigation; writing—original draft; writing—original draft. **Shin‐ichi Arimura**: Writing—original draft.

### Conflict of Interest

Patents related to the method described in this article are pending in Japan and the USA. The patents are held by the University of Tokyo.

## Data Availability

Data sharing is not applicable to this article as no new data were created or analyzed in this study.
